# Research and Remedies

**Published:** 1912-07-27

**Authors:** 


### RESEARCH AND REMEDIES.

### Arterial Tension During Digestion.

DR. LOEPER, in a recent number of *Les Archives des maladies de cœur*, reports some interesting observations with reference to the tension of the arteries during digestion. He finds that this tension undergoes three main variations during a meal, a primary or immediate rise closely following the ingestion; a secondary fall which takes place in from a quarter to three-quarters of an hour later; and, thirdly, after a few variations of which neither the causes nor the time can be accurately fixed, another elevation which reaches its maximum three hours or more after the meal. The primary rise he attributes solely to distension of the stomach, and it is the more marked the greater the bulk of the meal ingested. This is particularly to be noticed after large quantities of fluid have been taken, and it persists until the fluid has been passed on to the intestine. The secondary hypotension corresponds to the commencing activity of the gastric secretion, and it is more pronounced when the meal consists of substances such as salt and meat which powerfully excite gastric secretion. The delayed hypertension corresponds to intestinal distension and blood plethora. It is proportional to the abundance of digestive materials and to their irritative action on the vessels as well as to the rapidity of their elimination.

### Inequality of the Pupils in Pulmonary Disease.

SERGENT, in a recent number of the *Progrès Médical*, draws attention to the association between pupillary inequality and pleuro-pulmonary affections. When this inequality is fixed and constant occurring without associated symptoms and without any modification of the pupil reflexes, a pleural or pulmonary affection must be thought of, and in particular a tuberculous one. It may even happen that the oculo-pupillary syndrome observed in an old syphilitic case is quite independent of syphilis and brought about by the existence of a fibrosis of tuberculous origin, as is often seen in such patients. In such a case pupillary reflexes are normal. The author cautions the physician not to be in a hurry, even in case of a syphilitic, and more particularly if some pleuro-pulmonary affection has been diagnosed, to conclude from the presence of inequality of the pupils that the onset of tabes or general paralysis is imminent.

